# Application Status of Sacrificial Biomaterials in 3D Bioprinting

**DOI:** 10.3390/polym14112182

**Published:** 2022-05-27

**Authors:** Siyu Liu, Tianlin Wang, Shenglong Li, Xiaohong Wang

**Affiliations:** 1 Center of 3D Printing & Organ Manufacturing, School of Intelligent Medicine, China Medical University (CMU), No. 77 Puhe Road, Shenyang North New Area, Shenyang 110122, China; liusiyu4369@outlook.com (S.L.); tlwang@cmu.edu.cn (T.W.); slli@cmu.edu.cn (S.L.); 2 Center of Organ Manufacturing, Department of Mechanical Engineering, Tsinghua University, Beijing 100084, China

**Keywords:** three-dimensional (3D) bioprinting, sacrificial biomaterials, organ manufacturing, vascularization

## Abstract

Additive manufacturing, also known as three-dimensional (3D) printing, relates to several rapid prototyping (RP) technologies, and has shown great potential in the manufacture of organoids and even complex bioartificial organs. A major challenge for 3D bioprinting complex org unit ans is the competitive requirements with respect to structural biomimeticability, material integrability, and functional manufacturability. Over the past several years, 3D bioprinting based on sacrificial templates has shown its unique advantages in building hierarchical vascular networks in complex organs. Sacrificial biomaterials as supporting structures have been used widely in the construction of tubular tissues. The advent of suspension printing has enabled the precise printing of some soft biomaterials (e.g., collagen and fibrinogen), which were previously considered unprintable singly with cells. In addition, the introduction of sacrificial biomaterials can improve the porosity of biomaterials, making the printed structures more favorable for cell proliferation, migration and connection. In this review, we mainly consider the latest developments and applications of 3D bioprinting based on the strategy of sacrificial biomaterials, discuss the basic principles of sacrificial templates, and look forward to the broad prospects of this approach for complex organ engineering or manufacturing.

## 1. Introduction

Three-dimensional (3D) printing has developed rapidly during the past 20 years, and has undoubtedly proven to be an advanced technology that can revolutionize the manufacturing process of many industries, such as healthcare and medicine, environmental monitoring, aerospace, and automotive [1,2,3]. For biomanufacturing, one of the final goals is to design complex organs with vascular/neural networks to treat diseases with limited options, such as end-stage organ failure. So far, 3D bioprinting has achieved important milestones with respect to healthcare, including physiological device establishment, homogeneous/heterogeneous tissue construction, perfusable vascular network building, and implantable stent processing [4,5,6,7,8,9].

An important element of 3D bioprinting is the choice of printable materials. Natural polymers, such as collagen, alginate, chitosan, fibronectin, laminin, and their combinations, can mimic extracellular matrices (ECMs) with a large amount of water content [10,11,12]. For example, collagen is the main component of the natural ECMs that provide overall tissue stiffness and integrity [13,14], chitosan has an inherent antibacterial effect on many filamentous fungi and yeasts [15], fibronectin is a protein that dictates cell adhesion, spreading, migration, proliferation, and apoptosis [16,17,18], while laminin plays a key role in the structural formation and functional maintenance of the basement membrane [19]. An important property of most natural polymers is that they can dissolve in water and have good or excellent biocompatibilities. As early as 2005, the first cell-laden 3D bioprinting technology was created by Professor Wang in Tsinghua University with a series of collagen-derived gelatin-based natural hydrogels, such as gelatin, gelatin/alginate, gelatin/alginate/fibrinogen, gelatin/alginate/hyaluronate, for bioartificial tissue/organ manufacturing [20,21,22,23,24,25,26,27,28,29]. They are all rapid prototyping (RP) technologies, also named as additive manufacturing and solid freeform manufacturing. Nearly 20 years later, Muthusamy et al. used xanthan gum as a sacrificial biomaterial to print collagen for 3D tissue construction with capillary-like microvascular networks [30]. The cell-laden alginate/gelatin hydrogels have been used widely as a normal ‘bioink’ [31]. In addition, as natural hydrogels contain a lot of water, there is abundant space suitable for cell survival and growth, which is one of the important reasons that hydrogel has been widely used in 3D bioprinting as a biomaterial [32,33,34,35]. Many research groups have applied for patents based on 3D printing of natural hydrogels [36]. Natural hydrogels have been widely employed in 3D bioprinting and become the major substrates for bioartificial tissue/organ manufacture [37].

**Figure 1:**
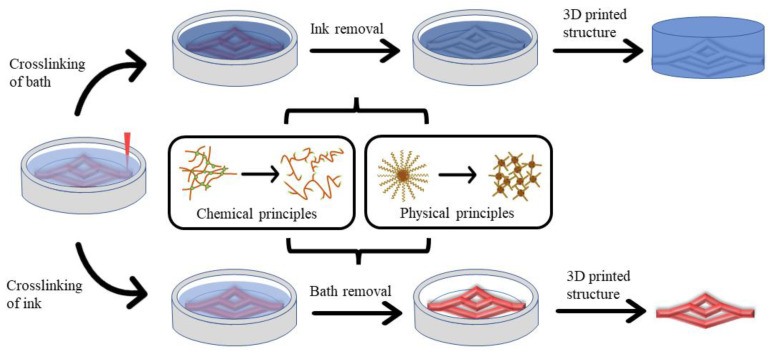
Overview of the main application scenarios of sacrificial biomaterials based on physical and chemical polymer crosslinking principles in 3D bioprinting. Top: The use of a physical crosslinked sacrificial polymer to form a tubular vascular network. Bottom: The use of a chemical crosslinked sacrifice polymer as a support bath to print the required vascular network.

Nevertheless, natural hydrogel-based 3D bioprinting has presented some obstacles for complex organ manufacturing. One of the obstacles is that most of the 3D printed hollow tubular structures collapse easily due to the low strength of the soft and dynamic hydrogels [18]. At this time, biomaterials that can be removed after printing are needed to achieve the precise construction of complex organ level structures. In recent years, biomaterials such as gelatin, Carbopol, Pluronic F127, and methylcellulose have been used widely in 3D bioprinting [7,38,39,40,41,42,43]. As a result, some soft hydrogels that were previously considered “unprintable” alone have been printed accurately based on the supporting suspensions composed of sacrificial biomaterials or ‘bioinks’ [38,44,45,46,47]. Sacrificial ‘bioinks’ are also named as flexible ‘bioinks’ or fugitive ‘inks’. The main feature of sacrificial biomaterials is that the relatively gentle and reversible crosslinking processes for polymers can be achieved through some physical or chemical principles, so that they can be “sacrificed” without harming the involved cells and desired biomimetic structures. Figure 1 summarizes the general applications of several sacrificial biomaterials based on physical and chemical crosslinking principles. As sacrificial biomaterials have a wide range of potential applications, some groups have published several reviews in this area. For instance, McCormack et al. summarized the advantages and development status of suspension printing [48], and Hu et al. summarized the advances of different crosslinking strategies for biomedical hydrogels [49]. In this review, we have more comprehensively sorted out the application scope of sacrificial biomaterials, and summarized the application states of sacrificial biomaterials based on physical and chemical principles, highlighting the advantages and disadvantages of these biomaterials in 3D bioprinting.

## 2. Sacrificial Biomaterials Based on Physical Principles

The physical crosslinking principle of the sacrifice biomaterials is based on the reversible interaction between molecules, mainly including critical temperature of the sacrificial polymers, the pH value of system, ultrasonic dispersion, and mechanical peeling [50,51,52,53,54]. The outstanding advantage of physical crosslinking is biomedical safety, because the processes do not use chemical crosslinking agents with potential cytotoxicity [55]. The sacrifice process also does not require complicated chemical reactions to achieve a reversible crosslinking and support material sacrificing. In Table 1, the advantages and disadvantages of several commonly used sacrificial biomaterials have been summarized.

There are many special characteristics of the sacrificial polymers based on the physical crosslinking principles. One of them is attributed to the ease of solubility in water. For example, gelatin and polyvinyl alcohol (PVA) structures can be removed quickly by immersing them in water [56]. The second one is temperature sensitivity. For example, Pluronic F127 solution can form gel at a certain concentration above 10 °C and liquefy when the temperature drops to 4 °C [9], while gelatin solution gels below 30 °C and liquefies when the temperature rises to 37 °C [57]. The third one is proper rheology. For example, gelatin solution can obtain better flowability after it is fabricated into gelatin microspheres via mechanical force [38,45]; in contrast, Carbopol is often used as a thickener with good rheological properties [44]. Because these properties make it easy to convert the polymers from solid to liquid, so they can be removed easily from the 3D printed constructs postprinting to achieve the purpose of sacrifice. Additionally, most of the sacrificial biomaterials have good biocompatibility (i.e., non-cytotoxicity) and degradability, so it is unlikely that any small amount of residual sacrificial biomaterials will affect the cell activity or integration.

### 2.1. Polyvinyl Alcohol

Polyvinyl alcohol (PVA), as a synthetic polymer, has received extensive attention in the past ten years due to its light transmission, water solubility, biocompatibility, hydrophilicity, and antiseptic properties [58,59], and been used widely in biomedical fields [60,61,62,63,64]. PVA solutions can form hydrogels through physical entanglement of the molecular chains through many weak non-covalent bonds, including hydrogen bonds and van der Waals. Compared with natural hydrogels, such as gelatin and alginate, PVA has better mechanical properties after fused deposition printing and is easily soluble in water or phosphate-buffered saline (PBS). PVA has several merits as a sacrificial material for organ 3D bioprinting.

As early as 2014, Jeffries et al. used PVA as a sacrificial material to construct microvessels in a 2D fibrous structure [65]. In 2016, Mohanty et al. proposed a method for preparing a double-hole scaffold with PVA [66]. They firstly poured polydimethylsiloxane (PDMS) into a 3D printed salt-containing PVA scaffold. After the PDMS was completely cured, the construct was immersed in water to dissolve the salt-containing PVA structure inside. This method could control the geometry and size of the channels, as well as the mechanical stiffness of the scaffold. The post-3D bioprinting cell experiment proved that the scaffold could provide a fine 3D microenvironment for cell growth and liver tissue formation. In 2020, Zou et al. printed a valentine heart scaffold with microfluidic channel networks using sodium alginate, agarose, and platelet-rich plasma (PRP) composite hydrogel as an ECM and PVA as a sacrificial structure. H9c2 cardiomyocytes and human umbilical vein endothelial cells (HUVECs) were seeded on the scaffold afterwards [66]. The results showed that the cell survival rate was above 90% after 28 days of culture. In the same year, Shimizu et al. used a 3D printed sacrificial PVA model to prepare a gelatin-based microchannel with a gradient generator and an in-channel micromixer [67]. The device used a tree-shaped network with a micro-mixer to generate a concentration gradient of the fluorescent dye solution to simulate the microenvironment in the body, such as chemical stimulation of vascular endothelial cells or mechanical stimulation caused by blood flow. In addition, the device could be used for basic biological research of cell response to external stimuli, as well as an in vitro platform for drug testing. In another study of theirs, a similar method was used to design a perfusion and stretchable gelatin-based microfluidic system that could simultaneously apply fluid shear stress and tensile stress to in vitro 3D tissue cultures [68]. HUVECs were successfully cultured in the 3D ECM like hydrogel perfused and stretched simultaneously with real-time imaging. In 2016, Li et al. reported a 3D branched network by printing water-soluble PVA filaments on a cylinder [69]. They embedded the 3D printed PVA structures in a gelatin solution and dissolved them in PBS solution after the gelatin structure was cross-linked to obtain a perfusible 3D channel. HUVECs were later injected into the perfusible channel and attached to the surface of the branched channel, proving that the remaining materials were nontoxic to cells. In 2021, Nagarajan et al. filled gelatin solution into a 3D printed PVA scaffold [70]. After the gelatin solution was cross-linked with glutaraldehyde, the construct was soaked in hot water to remove the sacrificial PVA. By changing the filling density of gelatin, gelatin scaffolds with different pore sizes and porosities could be constructed. Based on the perfusible vascular-like structure prepared by Li et al., an independent interconnected porous gelatin scaffold with a controllable structure was constructed. Experiments with MG63 osteoblasts showed that this scaffold was biocompatible and could achieved cell adhesion and proliferation without adding any cell adhesion molecules. This research provided a method to develop stable porous gelatin scaffolds with interconnected channels using sacrificial mold-assisted 3D bioprinting technology.

These studies demonstrated that 3D bioprinting microvessel networks using sacrificial PVA is possible, although the removal process of PVA is relatively slow, and the biodegradation rate of PVA is rather slow. Additionally, though PVA is nontoxic to cells, its biodegradation property is controversial. Reducing the residue of PVA in 3D printed structures or improving the biodegradability of PVA through chemical modification may be one of the research directions that needs continuous efforts.

### 2.2. Pluronic F127

**Figure 2:**
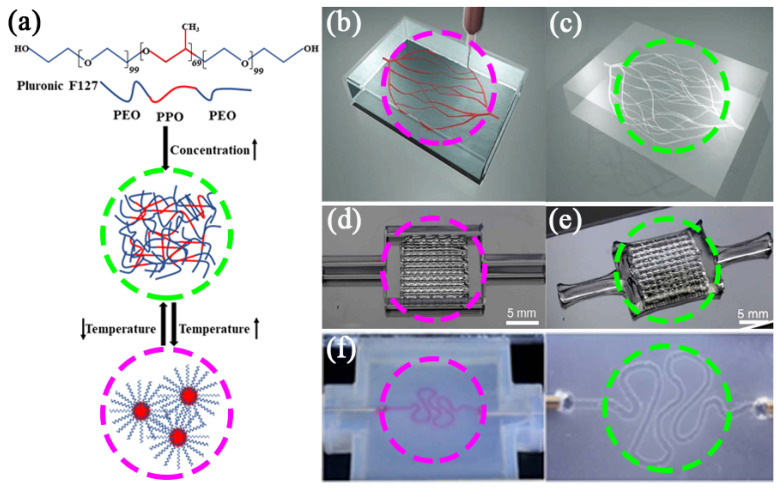
(**a**) Chemical structure of Pluronic F127 and the principle of temperature sensitivity. (**b**,**c**) Pluronic F127 as a sacrificial biomaterial for printing microvascular networks (scale bar = 10 mm [88]. Reprinted with permission from Ref. [88]. Copyright 2011, copyright Wu et al. (**d**,**e**) Pluronic F127 as a sacrificial biomaterial for printing interconnected channels [74]. Reprinted with permission from Ref. [74]. Copyright 2014, copyright Kolesky et al. (**f**) Pluronic F127 as a sacrificial biomaterial for printing perfusable renal tubules [73]. Reprinted with permission from Ref. [73]. Copyright 2016, copyright Homan et al.

Pluronic F127 is a synthetic non-ionic triblock copolymer, composing of hydrophilic poly(ethylene oxide) (PEO) and hydrophobic poly(propylene oxide) (PPO) blocks in the form of (PEO/PPO/PEO) [71]. This polymer is of particular interest in biomedical fields, owing to its triblock poloxamer structure with the alternating hydrophilic and hydrophobic segments, together with its thermal sensitivity and crystallinity [72]. The thermal sensitivity of Pluronic F127 is due to its solution concentration, which can be higher than its critical micelle concentration (CMC) at 4 °C. At this temperature, the hydrophobic PPO blocks are entangled with each other and become less soluble in water, resulting in the formation of micelles with dehydrated PPO cores and hydrated PEO shells, and realizing the gelation of the Pluronic F127 solution. This means that a high concentration of Pluronic F127 solution can form a viscoelastic gel when its temperature rises above 10 °C, and achieve a gel-to-sol transition when the temperature drops to 4 °C, allowing it to be removed under mild conditions [9,73,74]. Due to the temperature-sensitive characteristics of Pluronic F127, it is often used as a sacrificial template to form microscale vasculatures or blood vessel networks [75,76,77,78]. Additionally, Pluronic F127 has a wide range of viscosities and is easy to print without stressing the embedded cells through both extrusion-based and valve-based bioprinters [9,74,79,80]. Pluronic F127 is applied as a sacrificial biomaterial in bone regeneration [79,80,81], articular cartilage repairs [82,83,84,85,86,87], and vascular template construction [74,88,89,90], etc. It is an effective way to manufacture vascularized tissues (Figure 2a) [88].

As early as 2011, Wu et al. printed Pluronic F127 as a short-acting ‘ink’ in a medium composed of photocrosslinkable Pluronic F127 diacrylate (F127-DA) [88]. After photocrosslinking, the Pluronic F127 was removed at 4 °C to form a complex microvascular network (Figure 2b,c). The embedded microtubule network has shown some potential in tissue engineering and drug delivery. Although the material used (F127-DA) failed in relevant cell compatibility experiments because of its biological inertness and difficult biodegradation, this sacrificial template for preparing microtubule networks has come to the attention of scientists. In 2020, Zheng et al. prepared hydrogel microvascular tissues with hierarchical branched channels with a minimum feature inner diameter of 30 μm using electrohydrodynamic (EHD) inkjet printing technology, which is close to the physical scale of native capillaries [16]. They printed Pluronic F127 on a square PDMS substrate, and then cast flowable gelatin methacrylate (GelMA) solution (or cell-containing GelMA solution) at 37 °C onto it. After crosslinking GelMA by ultraviolet light, the entire structure was cooled to 4 °C to liquefy and remove the embedded Pluronic F127. HUVEC cells were finally seeded and cultured on the hierarchical branched channels. This method can integrate advantageous printing/casting processes and provides superior spatial resolution and flexibility of 3D structures. In 2014, Kolesky et al. printed 1D, 2D, and complex 3D vascular networks by embedding Pluronic F127 into GelMA (Figure 2d,e) and then removed the Pluronic F127 at 4 °C to form the networks [74]. By using the bioactive GelMA as ECM, it is possible to overcome the degradable shortcomings of Pluronic F127. After seeding HUVECs into the 2D vascular channel for 48 h, more than 95% of the cells retained viable and assembled into a nearly confluent layer. In subsequent studies, the researchers printed gelatin–fibrinogen bioink containing human mesenchymal stem cells (hMSCs) and human neonatal dermal fibroblasts (hNDFs) with Pluronic F127 were printed onto a perfusion chip which consisted of a silicone ‘ink’ [9]. After the Pluronic F127 structure was liquefied and removed at 4 °C, a vascularized tissue of more than 1 cm in thickness was formed, which could be perfused on the chip for a long time (>6 weeks). This approach has opened a new avenue for human tissue engineering. By combining bioprinting, 3D cells culture and organ-on-chip methods, Homan et al. demonstrated a customizable platform for manufacturing perfusable crimped 3D proximal tubules on a chip (Figure 2f) [73]. They defined the size and geometry of the tubules through CAD programming, used the enzymatic crosslinking of fibrinogen and gelatin as ECM and Pluronic F127 as sacrificial ‘bioinks’ to form hollow tubular structures, and promoted the adhesion of proximal tubule epithelial cells (PTECs) on the scaffolds to form a confluent layer, which could be maintained for more than 60 days. This 3D bioprinting method may be used to better simulate the microenvironment of cells in vivo, and has potential in drug screening, drug mechanism research, disease model establishment, and ultimately regenerative medicine.

Until now, the 3D printability of decellularized extracellular matrix (dECM) hydrogels has not been ideal. In 2019, Lewis et al. took advantage of the easily printed Pluronic F127 as a supporting structure to control the direction and geometry of engineered biliary trees [91]. After 3D printing, liver dECM containing mouse small bile duct cells (SV40SM44) were injected into the go-through pores to form the biliary tree-like structure. This 3D bioprinting technology, using sacrificial biomaterials to improve the printability of the biocompatible dECM, is used to allow co-cultivation of multiple cell types, such as hepatocytes and endothelial cells, and studying the cell–cell interaction mechanisms.

Though Pluronic F127 has been widely used as a sacrificial biomaterial in 3D bioprinting due to its thermal sensitivity characteristics, its shortcomings, such as biological inertness, have been clearly defined. Furthermore, the 3D bioprinting processes using sacrificial Pluronic F127 are time-consuming and may have limited usage for complex organ manufacturing with multiple vascular/neural networks.

### 2.3. Gelatin Microgel

**Figure 3:**
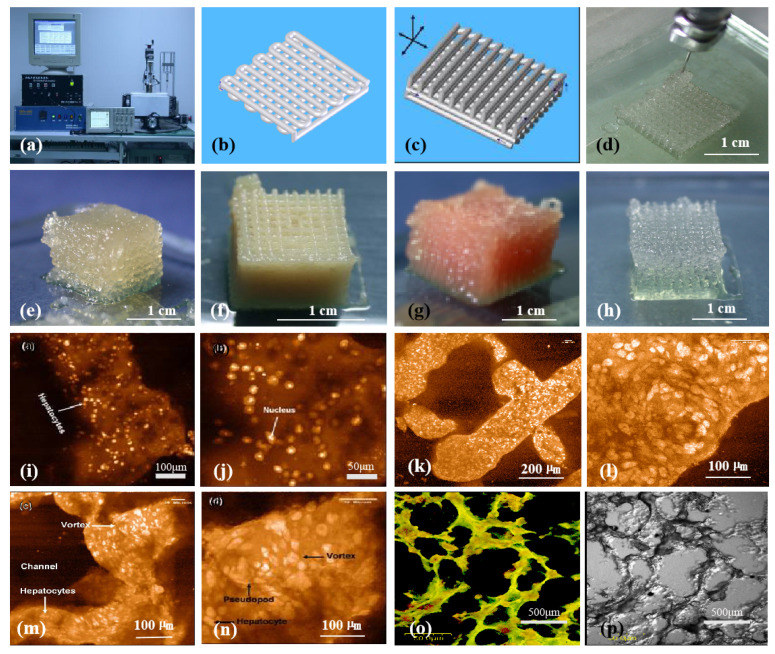
Three-dimensional (3D) bioprinting of chondrocytes, cardiomyocytes, hepatocytes, and adipose-derived stem cells (ASCs) into living tissues/organs using a pioneered 3D bioprinter made at Tsinghua University in Prof. Wang’s laboratory: (**a**) the pioneered 3D bioprinter; (**b**) schematic description of a cell-laden gelatin-based hydrogel being printed into a grid lattice using the 3D bioprinter; (**c**) schematic description of the cell-laden gelatin-based hydrogel being printed into large scaled-up 3D construct using the 3D bioprinter; (**d**) 3D printing process of a chondrocyte-laden gelatin-based construct; (**e**) a grid 3D construct made from a cardiomyocyte-laden gelatin-based hydrogel; (**f**) hepatocytes encapsulated in a gelatin-based hydrogel after 3D printing; (**g**) hepatocytes in a gelatin-based hydrogel after 3D printing; (**h**) a gelatin-based hydrogel after 3D printing; (**i**–**p**) hepatocytes in some gelatin-based hydrogels after certain periods of in vitro cultures [99].

**Figure 4:**
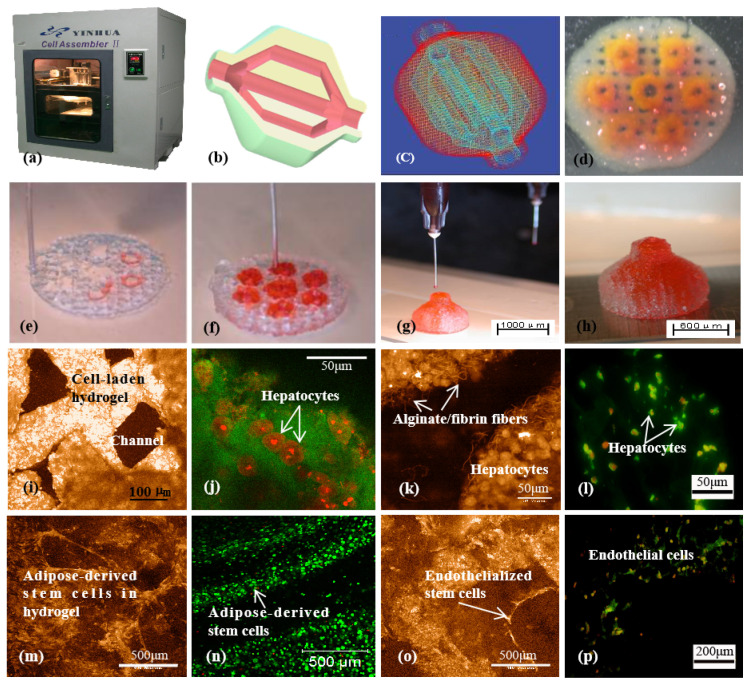
A large scaled-up 3D printed bioartificial organ with vascularized liver tissue constructed through the double-nozzle 3D bioprinter created at Tsinghua Unversity in Prof. Wang’s laboratory: (**a**) the double-nozzle 3D bioprinter; (**b**) a computer-aided design (CAD) model containing a branched vascular network; (**c**) a CAD model containing the branched vascular network; (**d**) a few layers of the 3D bioprinted construct containing both adipose-derived stem cells (ASCs) encapsulated in a gelatin/alginate/fibrin hydrogel and hepatocytes encapsulated in a gelatin/alginate/chitosan hydrogel; (**e**–**h**) 3D printing process of a semi-elliptical construct containing both ASCs and hepatocytes encapsulated in different hydrogels; (**i**–**l**) hepatocytes encapsulated in the gelatin-based hydrogels after 3D bioprinting and different periods of in vitro cultures; (**m**–**p**) ASCs encapsulated in gelatin-based hydrogels after 3D bioprinting and different periods of in vitro culture as well as growth factor induction [99].

Gelatin is a derivative of collagen and an excellent biomaterial with thermoreversible properties [92,93,94]. It can dissolve in water and form an aqueous solution at 37 °C or higher, which turns to a gel state physically at a temperature below 30 °C [57,95]. With a lot of polar groups, such as amino carboxyl groups, in its molecules, gelatin can absorb much more water in a hydrogel compared to its own weight. In gelatin solution, the main bodies of the linear polymers adhere to each other. As the temperature decreases, the thermal energy of the molecules decreases, the van der Waals force physical cross-links the molecular chains to form a homogeneous structure, which prevents the molecules from moving with each other, and finally the molecules lose their mobility. After gelation, a large number of water molecules are firmly adsorbed by the polar groups of the molecular chains, resulting in the formation of tough and elastic gels. Compared with collagen, gelatin has no antigenicity with better biocompatibility and lower price. Meanwhile, it also inherits the properties of collagen to promote cell adhesion. Compared with the abovementioned Pluronic F127, the advantage of gelatin for organ manufacturing is that it has excellent bioactivities with rapid biodegradation rates [96,97,98]. When it is implanted in vivo, there are no biodegradable residues affecting the biochemical cycles. So, gelatin has been widely used in direct bioprinting technologies from the very beginning as an ideal natural biomaterial (Figure 3 and Figure 4). Particularly, gelatin has also been widely used as a sacrificial biomaterial in 3D bioprinting because of its temperature-sensitive properties [96,97,98].

**Figure 5:**
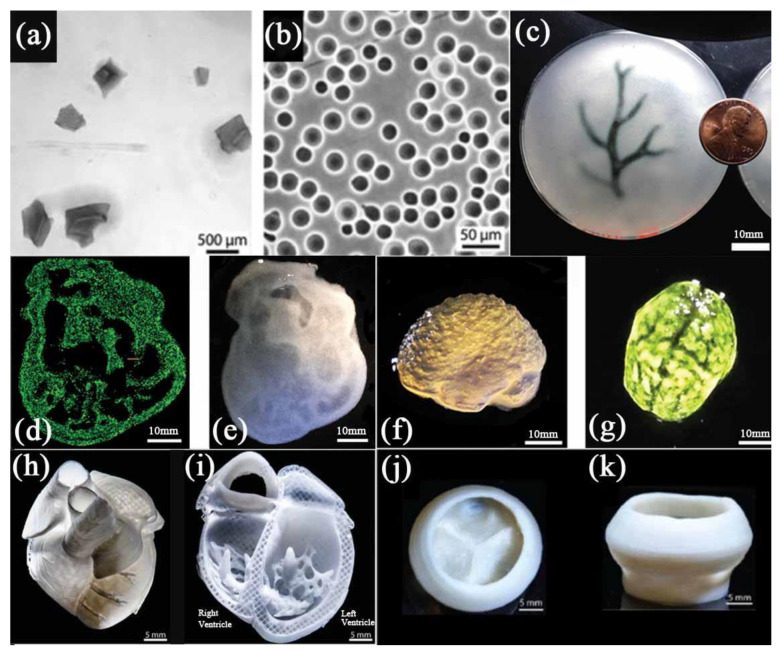
(**a**) Gelatin particles prepared by mechanical force. (**b**) Gelatin microspheres prepared by coacervation method. (**c**) A schematic diagram of the arterial tree printed in a gelatin microgel supporting bath. (**d**) A cross section of the 3D printed heart in fluorescent alginate. (**e**) Dark field image of the 3D printed heart. (**f**) Side view of the brain printed with alginate. (**g**) The top view of the 3D printed brain. (**h**) MRI-derived 3D human heart scaled to neonatal size. (**i**) FRESH-printed collagen heart. (**j**,**k**) Top and side views of the FRESH-printed collagen heart valve with barium sulfate added for X-ray contrast. Scale bars: 10 mm in (**c**) and 1 cm in (**d**–**g**); (**a**,**c**,**d**,**f**,**g**) are from reference [45], Reprinted with permission from Ref. [45]. Copyright 2015, copyright Hinton et al. (**b**,**h**,**i**,**j**,**k**) are from reference [38], Reprinted with permission from Ref. [38]. Copyright 2019, copyright Lee et al.

In 2015, Hinton et al. reported a novel 3D bioprinting technology called freeform reversible embedding of suspended hydrogel (FRESH) pertinent to gelatin [45]. They first crushed the gelatin into particles with a diameter of about 65 μm by mechanical force to form a gelatin microgel (Figure 5a). This makes gelatin microparticles, like Bingham plastic, behave as a rigid body under low shear stress, while as a viscous fluid under high shear stress. This means that when a needle-shaped nozzle passes through the gelatin bath, there is almost no mechanical resistance, but the hydrogel can be squeezed out of the nozzle and deposited in the bath. The researchers printed complex structures, such as the coronary artery tree, a chicken heart and a human brain, in the gelatin microgel support bath successfully (Figure 5c–g). After 3D bioprinting, the gelatin supporting bath was removed gently at 37 °C. The obtained structures showed that the FRESH method can be used to print complex 3D structures with acceptable resolution. In further research, the researchers developed a coacervation approach to prepare regular spheres with a diameter of about 25 μm, enabling the 3D bioprinting accuracy to reach about 20 μm (Figure 5b) [38]. A combination of human stem cell-derived cardiomyocytes and collagen was employed as a ‘bioink’ in the gelatin supporting bath. Several 3D printed organ models, such as a ventricle, a tricuspid valve, and even a newborn-scale human heart, were obtained by removing the support bath (Figure 5h–k).

Similarly, FRESH technology was also used by other groups. For example, Spencer et al. used water solvents containing Ca^2+^ ions to dilute gelatin particles in order to achieve the purpose of improving printing resolution [100]. They printed electric conductive biomaterials in the support bath, which not only could print complex structures, but also showed high cellular viability when loaded with C2C12 cells (muscle myoblasts). Electric conductive biomaterials contribute to the function of the heart in the body. Their research has a good application prospect for complex conductive and cell-loaded hydrogels in cardiac tissue engineering [101]. Choi et al. also crushed gelatin into particles by mechanical force, but the difference is that they added poly (acrylic acid) as an active co-solvent [102]. They successfully printed the layered structure of vascularized muscle tissue in order to treat volumetric muscle loss. The printed muscle tissue showed an improved new formation of muscle fiber, vascularization, and innervation. Jeon et al. printed photocrosslinkable oxidized-methacrylated alginate (OMA) microgels with hMSCs encapsulated into a support bath composed of gelatin microgels [103]. The 3D printed structures were then transferred to the preheated Dulbecco’s Modified Eagle’s Medium (DMEM) (38 °C) to remove the gelatin bath. Using this technique, biologically structures, such as the femur, skull, and ear, could also be printed with the hMSCs-loaded microgel ‘bioinks’ through squeezing.

Especially, sacrificial gelatin can improve the porosity of 3D bioprinted tissues. More and more studies have shown that the size, connectivity, and geometry of pores in a 3D construct directly affect the incorporated cell behaviors, such as proliferation, migration, infiltration, and ECM deposition [32,33,34,35]. Therefore, controllable adjustment of porosity in a 3D construct to enhance cell function has become one of the important goals for most of tissue engineers and organ manufacturers [104,105]. Generally, the porosity of a gelatin-based hydrogel can be increased by reducing the concentration, but it can significantly reduce the viscosity (printability) of the ‘bioinks’. To achieve the printability of the ‘bioinks’, researchers have exploited various strategies, such as the usage of the gelatin microgels and the addition of the GelMA microgels [106,107]. The resultant porosity of these ‘bioinks’ ranged from 0.20 ± 0.02 to 0.57 ± 0.06 (100:0 and 40:60, respectively) after removing the sacrificial gelatin microgel, exceeding the theoretical limit of microgels without the sacrificial components. HUVECs maintained viability for up to 7 days during in vitro culture, showing migration to the 3D printed structure to a certain extent, which depended on the enhanced porosity of the ‘bioinks’. Parallelly, Konka et al. added gelatin microspheres as a sacrificial biomaterial to the self-setting hydroxyapatite ‘inks’ [108]. The partial dissolution of the gelatin microspheres caused spherical pores to be formed on the surface of the 3D printed fibers, which increased the porosity of the fibers from 0.2% to 67.9%. The concave pores formed in the 3D printed calcium phosphate scaffold were expected to provide a suitable environment for cell effective adhesion and proliferation. Moreover, the residual gelatin could provide more elastic behavior for the scaffold, creating another advantage of the 3D printed constructs.

Thus, gelatin-based particles and their supporting hydrogel baths are a typical extension of the traditional gelatin-based 3D bioprinting strategies, which may play a special role in complex organ manufacturing with both vascular and neural networks.

### 2.4. Carbopol

**Figure 6:**
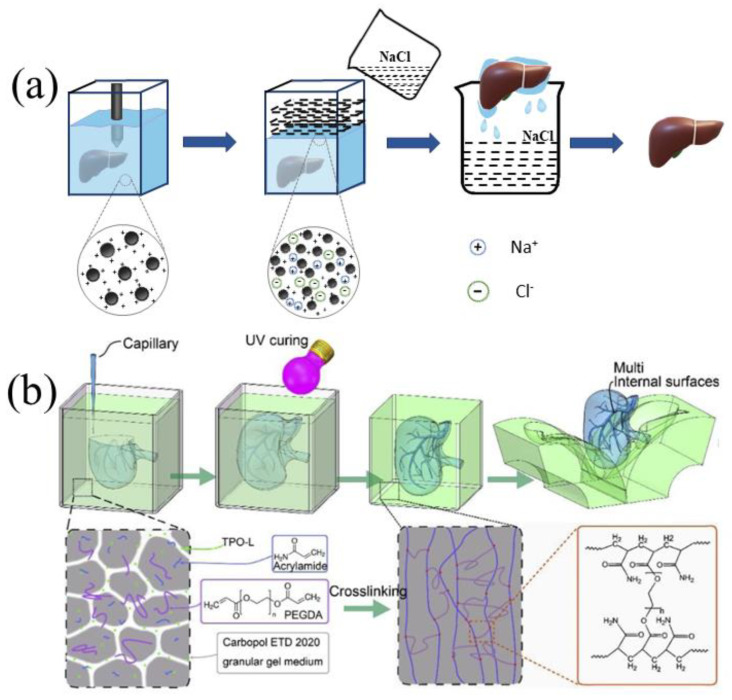
(**a**) Changing the rheology of the Carbopol bath by shielding the electrostatic repulsion between Carbopol ions to remove the Carbopol support bath. (**b**) After photo-crosslinking, the Carbopol support bath was removed by mechanical peeling [43], Reprinted with permission from Ref. [43]. Copyright 2020, copyright Zhao et al.

Carbopol is a high molecular weight poly(acrylic acid) with low internal crosslinking density, which gives Carbopol gel some particular characteristics [109]. As a rheology modifier, aqueous solutions of Carbopol can be neutralized and thickened by adding NaOH and KOH at low doses. Like gelatin microgels, Carbopol microgel could provide a better solution to the problems of the extrusion-based 3D bioprinting of soft materials [44,110]. In some cases, Carbopol can be regarded as a kind of swelling microgel suspension, and its swelling property is the result of electrostatic interaction, which is very sensitive to the ionic properties of the suspension medium. Physiological saline, such as NaCl solution, has a strong influence on the rheology of Carbopol suspension. The electrostatic repulsion between the Carbopol microgels in the initial suspension causes the Carbopol microgel to swell. The addition of NaCl solution or Dulbecco’s phosphate-buffered saline (DPBS) can effectively shield the electrostatic repulsion, shrink the Carbopol microgels, and fluidize the suspension, with reduced yield stress and viscosity (Figure 6).

Due to both Carbopol microgels and alginate molecules reacting with Ca^2+^, researchers used a filament extrusion-based two-step gel method to obtain 3D alginate structures [111]. That is, after the gelatin–alginate structure was printed in the Carbopol bath, the Carbopol bath and gelatin structure were removed sequentially while the alginate structure was crosslinked in CaCl_2_ solution at 37 °C. The researchers tested three biological solutions, namely, DMEM, 0.9% (*w*/*v*) sodium chloride solution, and 10% (*w*/*v*) deionized water sucrose solution, to rinse and facilitate removal of the Carbopol gel bath. Compared with DMEM and sucrose solution, the yield stress of the Carbopol bath decreased from about 19 Pa to 2 Pa after immersion in sodium chloride solution, and the zero-shear-rate viscosity reduced from about 1100 Pa·s to 300 Pa·s.

As stated above, Carbopol can be regarded as a suspension of swollen microgels. The swelling is the result of electrostatic interaction and is therefore sensitive to the ionic properties of the suspension medium. Physiological saline solutions such as sodium chloride solution have a great influence on the rheological properties of the Carbopol suspension. It is electrostatic repulsion between the Carbopol microgels in the initial suspension that causes the Carbopol microgels to swell. The excessive ions introduced by the sodium chloride effectively shield the electrostatic repulsion, which causes the Carbopol microgel to shrink and the suspension to become more fluidic with the decrease of the yield stress and viscosity. Because the DMEM solution contains non-ionic components and salts to obtain the same osmotic strength as the physiological saline, the physiological saline solution has a slightly higher ionic strength than the DMEM. It is the most effective way to use 0.9% (*w*/*v*) sodium chloride solution to clear the remaining Carbopol before the ionic crosslinking of the printed structures [111,112,113,114].

In 2020, Lee et al. printed photocrosslinkable GelMA and methacryloyl-substituted recombinant human tropoelastin (MeTro) composite hydrogel onto the Carbopol suspension medium [112,113,114]. After photopolymerizing the printed structure, they liquefied Carbopol bath by adding Dulbecco’s phosphate-buffered saline (DPBS) with monovalent cation to release the printed structure. The printed structure was then cultured at 37 ℃ to remove the residual Carbopol and gelatin. The 3D printed vascularized heart tissue exhibited endothelial barrier function with spontaneously beating cells, which are important functions of the heart tissues in the body. Furthermore, the printed construct caused minimal inflammation and was shown to be biodegradable in vivo when implanted subcutaneously in rats. These results certify the potential of using MeTro/GelMA composite hydrogel to print complex 3D functional heart tissues in a Carbopol support bath. In the same year, Ning et al. evaluated the effect of different concentrations of Carbopol solution as a support bath on the fidelity of printing [115]. They found that a 0.4% concentration of Carbopol solution was optimal, because it could firmly retain the printed structure with high fidelity, while it would not interfere with or block the printing of GelMA hydrogel. In addition, they were the first to explore the influence of the Carbopol bath on the penetration of ultraviolet rays. The results showed that the Carbopol bath did hinder the penetration of ultraviolet rays, resulting in the decrease of the mechanical properties of the hydrogel, which confirmed that the penetration of ultraviolet rays is an important factor to consider in the future design and manufacture of related structures.

In 2019, Ozbolat et al. first used Carbopol gel as sacrificial biomaterial for channel formation to fabricate microfluidic devices [116]. They printed channel templates of Carbopol hydrogel in the frames of silicone elastomer (SE 1700) and PDMS (Sylgard 184), using it to form a microfluidic structure by removing a sacrificial Carbopol template with deionized water. Two types of endothelial cells (primary HUVECs and endothelial cell lines, or BMECs) were perfused into the device and both were seen to adapt to gradually changing flow conditions by remodeling their cytoskeleton, cell shape, and nuclear orientation. The results proved that it is possible to fabricate infusible microfluidic devices using the versatility of Carbopol as a sacrificial bioink [117].

In contrast to the method of reducing viscosity of Carbopol, Zhao et al. mixed Carbopol with acrylamide, polyethylene glycol diacrylate, and TPO-1 (as a photoinitiator) to form a photo-crosslinked support medium with shear-thinning behavior and yield stress characteristics [43] (Figure 4b). After the printing was completed, the Carbopol support bath was crosslinked into a solid by ultraviolet radiation, and then the solid Carbopol structure was mechanically peeled off to obtain the printed structure. Compared with other studies, the Carbopol microsphere gel in this work not only served as a support medium for models or objects, but also became the object itself after crosslinking and acted as a barrier. To a certain extent, it could be regarded as the inverse process of the conventional EMB3D bioprinting strategy. Compared with some other organ manufacturing models based on the fused deposition modeling and casting process, the printing method of this study has greatly simplified the model editing process, providing new ideas and directions for suspension organ 3D bioprinting or embedded organ 3D bioprinting.

## 3. Sacrificial Biomaterials Based on Chemical Principles

Covalent bonds are usually formed among polymer chains in chemically crosslinked hydrogels, and most of the connection bonds are stronger compared to those of physically crosslinked hydrogels. Up to now, several chemical crosslinking methods have been reported, including free radical polymerization-induced crosslinking, enzyme-induced crosslinking, Diels–Alder click reaction, Schiff base formation, oxime formation, and Michael-type addition [118]. Compared with physically cross-linked hydrogels, chemically crosslinked hydrogels generally exhibit enhanced structural stability, excellent mechanical properties and adjustable degradation behavior under physiological conditions [118].

The commonly selected reverse crosslinking reactions for sacrificial biomaterials based on chemical principles are relatively mild (e.g., alginate [119]). Some of the reactions can be substituted by chemical modification (e.g., hyaluronic acid (HA) [120,121]). The advantage of sacrificial biomaterials based on chemical principles is that ideal mechanical and rheological properties of the supporting structures can be obtained. In some cases, the sacrificial biomaterials applied through chemical crosslinking principles can play a better supporting role than those through physical crosslinking principles. Some sacrificial biomaterials based on chemical crosslinking principles also have good biocompatibility and degradability, to ensure biosafety even if there are trace residues. Several commonly used sacrificial biomaterials based on chemical crosslinking principles are summarized in Table 1.

### 3.1. Alginate

**Figure 7:**
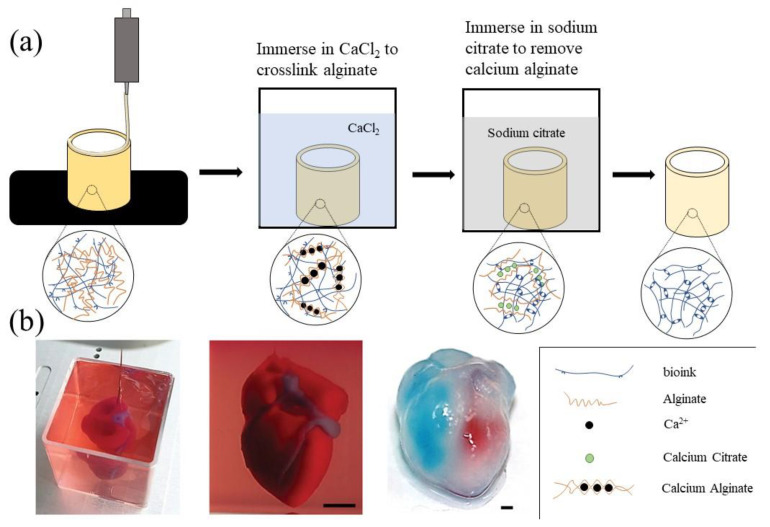
(**a**) Schematic diagram of the application of alginate as a sacrificial biological material in 3D bioprinting. (**b**) 3D bioprinting a heart structure in a suspension bath composed of alginate [127], Reprinted with permission from Ref. [127]. Copyright 2019, copyright Noor et al.

Sodium alginate is a natural polysaccharide isolated from brown algae. It is a linear anionic random block copolymer composed of BD-mannuronic acid (M unit) and a-L-guluronic acid (G unit) [122]. When sodium alginate solutions are exposed to divalent calcium ions, Ca^2+^ can replace the Na^+^ in sodium alginate to form calcium alginate gels. Some calcium chelating agents, such as sodium citrate and ethylenediaminetetraacetic acid (EDTA), can capture Ca^2+^ in the calcium alginate gels and mildly liquefy the solidified alginate structures, which provides the chemical basis of sodium alginate as a sacrificial biomaterial for 3D bioprinting [123,124,125] (Figure 7a). As a chemical sacrificial biomaterial, alginate is particularly effective in preparing protein-based microfibers for cell proliferation [126].

In 2020, Wang et al. printed sodium alginate into a CaCl_2_ (100 mM) solution to form tubular structures and poured suspension ECM prepolymer to encapsulate the calcium alginate structures [119]. After crosslinking the suspension ECM prepolymer and removing the calcium alginate structures using sodium citrate solution, a microfluidic pump was used to inject the culture medium into the channel to form a microfluidic network, which could control the soluble chemical environment in the 3D cultures and establish a flexible platform for a wide range of biomedical applications. In 2021, John et al. fabricated 3D (ε-caprolactone) (PCL)/gelatin (1:1) nanofiber aerogels with patterned macrochannels and anisotropic microchannels via freeze-casting 3D-printed sacrificial templates [127]. They added the short nanofiber suspension to a copper (Cu) mold containing a 3D printed alginate grid, then the aerogel was soaked in EDTA solution to remove the sacrificial alginate template. In vitro and in vivo studies have shown that nanofiber aerogels with patterned macrochannels could provide an ideal matrix for cell infiltration and host tissue integration. The construction of both macrochannels and microchannels in an object is an ideal method to manufacture bioartificial organs for drug screening and organ restoration [21].

In 2017, Compaan et al. combined sodium alginate as a sacrificial biomaterial with silk fibroin as ECM, and used a two-step gel method to make up for the shortcomings of slow crosslinking of silk fibroin. In the first step, the printed structure was immersed in a CaCl_2_ solution to gel the sodium alginate instantaneously [124]. In the second step, the printed structure was immersed in a hydrogen peroxide bath, and the silk fibroin embedded in the calcium alginate construct was enzymatically crosslinked for covalent gelation. Alginate provided structural definition during the printing process, which could be removed gently by sodium citrate or EDTA. The obtained structures showed that the inkjet-based printing based on the two-step gelation strategy was an effective 3D bioprinting method, while the removal process of alginate from the printed constructs through immediate citrate treatment had little effect on the related cell morphology or long-term metabolic activity. It proved that alginate is suitable for 3D bioprinting as a sacrificial suspension support bath.

Because alginate has good biocompatibility, its suspension medium can maintain cell viability and also serves as ECM [21]. Noor et al. used alginate microparticles in xanthan gum-supplemented growth medium to form a suspension bath, which could undergo safe enzymatic or chemical degradation for extraction [128]. A bioartificial heart was extracted from the suspension bath, and the integrity of the ventricle was proved by injection of blue dyes, red dyes, and fluorescent staining of actin. Its mechanical properties were also found to be like that of a rat heart. All these results have certified the feasibility of printing complex organ structures in an alginate suspension bath (Figure 7b). Jeon et al. developed a support bath composed of degradable photo-crosslinked OMA microgels [47]. The self-healing properties of the OMA microgel supporting medium allowed direct printing of hMSCs into the microgel support medium, exhibiting similar properties to Bingham plastic fluids. This was the first report of a bioprinting strategy using single cells as ‘bioinks’. As the size of the microgel decreases, it is possible to construct complex organ structures with vascular networks, due to the medium shear thinning characteristics when the needle is moving, the self-healing characteristics without external strain, and the limited diffusion characteristics when printing cells into its pores. In addition, the OMA microgels could be removed from the structure by simple agitation or spontaneous degradation, and the cultured structure could be easily obtained from the alginate microgel support medium without being damaged. This universally applicable 3D bioprinting strategy makes it possible to print isolated cells without the need for other biomaterial carriers in ‘bioinks’, allowing the generation of engineered tissues based on bionic cell condensation with multiple cell types and controlled spatial locations.

Furthermore, Tan et al. developed a recyclable calcium alginate microgel bath composed of alginate, Tween 20, CaCl_2_, and glycerin [129,130]. This alginate microgel bath had not only biocompatibility and printability, but also thermal stability, which enabled it to withstand the high-temperature curing of silicone ink. A recycling step to restore the original state of the degraded microgels with calcium chloride solution was exploited, which can effectively reduce the waste of the microgel bath.

### 3.2. Modified Hyaluronic Acid

**Figure 8:**
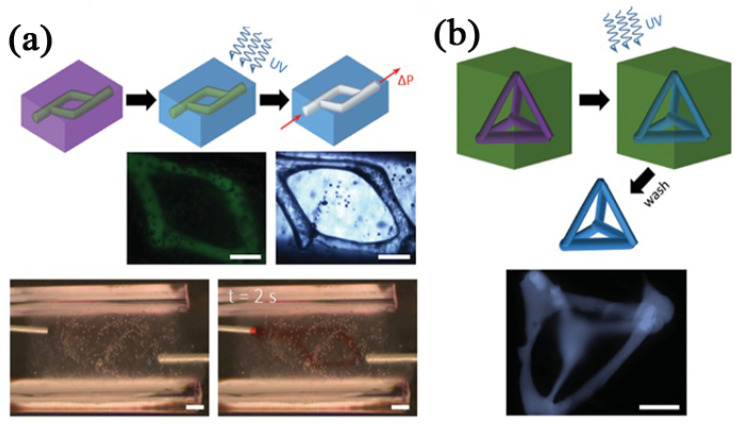
(**a**) 3D bioprinting a branched channel. After the 3D printed ‘bioink’ was UV crosslinked, a flow driven by pressure caused the supporting suspension to be removed, leaving the branched channel structure [120]. (**b**) 3D bioprinting a self-supporting structure. After the 3D printed ‘bioink’ was crosslinked by UV irradiation, the supporting suspension was dissolved by excess β-CD [120]. Scale bar 500 μm. Reprinted with permission from Ref. [120]. Copyright 2015, copyright Highley et al.

Hyaluronic acid (HA) is a linear polysaccharide composed of alternating units of repeating disaccharides, β-1,4-D-glucuronic acid-β-1,3-N-acetyl-D-glucosamine. It is a ubiquitous immune neutral polysaccharide in the human body. From the vitreous body of the eye to the ECM of cartilage tissue, HA can be found throughout the body as a highly hydrated polyanionic macromolecule. The HA has been widely used in biomedical research [131]. As an important part of ECM, HA has special meanings in organ 3D bioprinting with respect to cell signal transduction, wound repair, tissue/organ morphogenesis and energy metabolism [132,133]. When different chemical modification treatments are applied to HA, it can be used as sacrificial biomaterial for 3D bioprinting, due to the affinity and biocompatibility of chemically modified HA [109,110]. For instance, Burdick’s team developed a supramolecular hydrogel based on modified HA with either adamantane (Ad) or β-cyclodextrin (β-CD) (Ad-HA and CD-HA, Figure 8a), which could assemble rapidly upon mixing of Ad-HA and CD-HA through intermolecular guest–host bonds (between Ad and β-CD moieties). They used methacrylate modified Ad–HA or CD–HA (Ad- MeHA or CD-MeHA) as the supporting gel and a mixture of Ad-HA and CD-HA as sacrificial biomaterial to print a tetrahedral structure. Through the flow of needles inserted into the gel at the entrance and exit of the channel structure, the 3D printed ‘bioink’ was removed by excess β-CD, which competes with CD–HA to bind with Ad–HA, leaving an open channel with a printed pattern and size (Figure 8b).

In 2016, Shi et al. developed a new ‘bioink’ using bisphosphonic acid functionalized hyaluronic acid (HA-BP) and Ca^2+^ ions [134,135]. The HA-BP derivative was mixed with calcium chloride solution to quickly form a hydrogel. In their study, 1-ethyl-3-(3dimethylaminopropyl) carbodiimide (EDC)-mediated coupling reaction was used to introduce acrylamide (Am) groups into the HA-BP derivatives to create bifunctional AmHA-BP derivatives in order to enhance the mechanical properties for 3D bioprinting. When the obtained physical Am-HA-BP•Ca^2+^ hydrogel served as a supporting bath, the BP•Ca^2+^ coordination bonds could be cleaved in a slightly acidic environment, and then the 3D printed structure was separated from the supporting groove. The advantages of this ‘bioink’ were bio-friendliness and rheologic. Thick composite hydrogel structures were obtained through the media adsorbable on the living cells [136,137,138,139].

In 2020, Thomas et al. reported a method of using a stereolithography 3D printer to manufacture complex blood vessels [140]. They selected methacrylated hyaluronic acid (HAMA) containing HUVECs as the sacrificial biomaterials, and the HAMA molecules could be degraded by precise control of hyaluronidase. The released HUVECs from the sacrificial HAMA biomaterials could rearrange and adhere on the channel wall to form vascular-like structures.

Although HA and its derivatives have the characteristics of biological friendliness and self-healing properties, which are suitable for cell adhesion and growth, the chemical modification processes to realize the sacrificial function are very complex, which has become the major bottleneck restricting the application of these as sacrificial biomaterials. Once the chemical modification of the HA becomes simple, it will open some additional avenues for organ manufacturing with these sacrificial biomaterials.

**Table 1:** Advantages and disadvantages of several commonly used sacrificial biomaterials for organ manufacturing.

Biomaterials	Principle	Bioprinting Method	Advantage	Deficiency	Application	References
PVA	Physical	Fused deposition modeling	Biocompat ibility; water soluble	High printing temperature; not bioactive	Microtubule network	[56,65,66,67,68,69,70,71,72]
Pluronic F127	Physical	Extrusion 3D printing	Bio-friendly; easy to remove; shear-thinning	Not bioactive	Microtubule network	[9,16,73,74,75,76,77,78,79,80,81,82,83,84,85,86,87,88,89,90,91,92,93,94,95]
Gelatin	Physical	Extrusion 3D printing	bio-friendly; easy to remove; yield stress fluid behavior	Complex manufact uring process	Suspension medium/ increased porosity/sup port ing structure	[38,45,96,97,98,99,100,101,102,103,104,105,106,107,108]
Carbopol®	Physical	Extrusion 3D printing	bio-friendly; high transparency; lower dosage; easy to remove	-	Suspension medium	[43,109,110,111,112,113,114,115,116,117]
Alginate	Chemical	Inkjet 3D printing/ extrusion 3D printing	Bio-friendly; shear-thinning	Difficult to remove	Tubular tissue/supporting structure/ suspension medium	[47,118,119,120,121,122,123,124,125,126,127,128,129,130]
Modified hyalur- onic acid	Chemical	Extrusion 3D printing/stereo lithography 3D printing	Bio-friendly; shear-thinning; self- recovery	Difficult to synthesize; difficult to remove	Suspension medium	[119,120,131,132,133,134,135,136,137,138,139,140]

## 4. Challenges and Prospects

In recent years, the strategy of using sacrificial biomaterials to support 3D bioprinting technologies has shown some potential in overcoming the collapse phenomena in tubular vascular structure construction. Several groups of soft material and suspension printing performs have been set up with the layer-by-layer RP building processes. However, this strategy still encounters some challenges for complex organ manufacture. Firstly, many organs in nature have layered vascular networks, which means that printing a complete organ requires different biomaterials with different physiological functions. More combined multi-nozzle 3D printers should be explored to simultaneously print different biomaterials (including the sacrificial biomaterials required). Secondly, different sacrificial biomaterials have some defects, which may not have good printing performance, biocompatibility, and mechanical properties. In future research, different sacrificial biomaterials can be combined together to obtain ideal geometrical properties. Additionally, new bioactive sacrificial biomaterials can be developed according to the custom requirements. Some simple modification methods of bioactive materials can be sought, which can achieve simple removal procedures, and provide sufficient mechanical properties for the 3D constructs. Thirdly, unlike traditional direct organ 3D bioprinting, the suspended printing tip can be printed layer-by-layer at different positions of the medium, which can greatly improve the printing speed. However, the accuracy and resolution of suspended printing is inferior to that of traditional direct organ 3D bioprinting, and the highest line-width resolution of suspended printing is about 100 μm, which is much larger than the traditional 10 μm [94,141]. This limitation of printing accuracy has a great influence on the formation of the capillary networks. One of the main reasons for this limitation is the size of the inner diameter of the printing needle. When 3D bioprinting is carried out in suspension medium, the length of the needle determines the height of the printed object [142]. In the printing process, the needle is too long or too thin, which means it is easy to deform, causing the end of the printing needle to be unable to move accurately according to the predetermined position. It is also challenging to print more than three types of cells/ECMs in a very small space or compartment. Especially, the increase in printing resolution can cause the printing time to increase exponentially with the current 3D bioprinting technologies [143,144].

**Figure 9:**
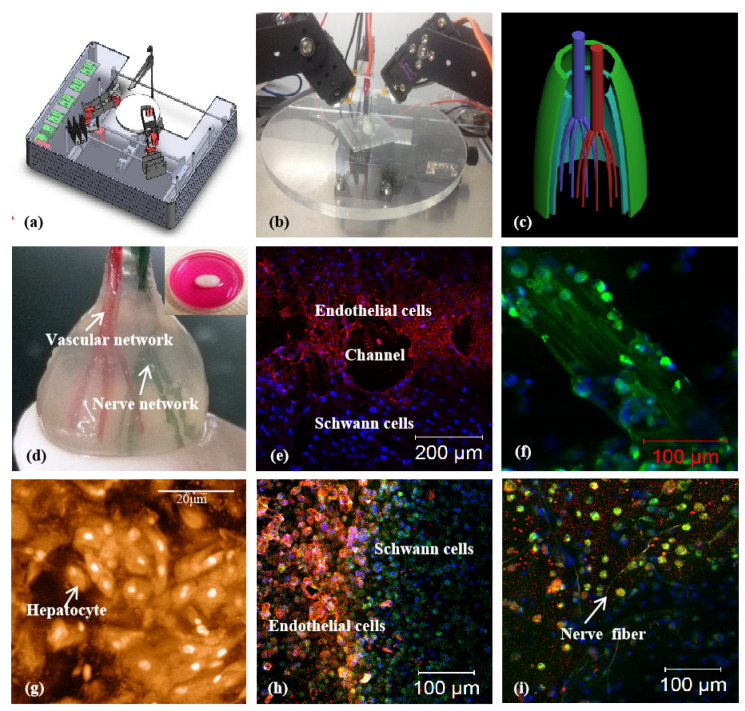
A combined four-nozzle organ three-dimensional (3D) bioprinting technology created at Tsinghua Unversity in Prof. Wang’s laboratory in 2013 [99]: (**a**) equipment of the combined four-nozzle organ 3D bioprinter; (**b**) working state of the combined four-nozzle organ 3D printer; (**c**) a computer aided design (CAD) model representing a large scaled-up vascularized and innervated hepatic tissue; (**d**) a semi-ellipse 3D construct containing a poly (lactic acid-co-glycolic acid) (PLGA) overcoat, a hepatic tissue made from hepatocytes in a gelatin/chitosan hydrogel, a branched vascular network with fully confluent endothelialized adipose-derived stem cells (ASCs) on the inner surface of the gelatin/alginate/fibrin hydrogel and a hierarchical neural (or innervated) network made from Schwann cells in the gelatin/hyaluronate hydrogel, the maximal diameter of the semi-ellipse can be adjusted from 1 mm to 2 cm according to the CAD model; (**e**) a cross section of (**d**), showing the endothelialized ASCs and Schwann cells around a branched channel; (**f**) a large bundle of nerve fibers formed in (**d**); (**g**) hepatocytes underneath the PLGA overcoat; (**h**) an interface between the endothelialized ASCs and Schwann cells in (**d**); (**i**) some thin nerve fibers.

At present, the formation of a tubular networks mostly relies on the self-assembly of various vascular cells. The limitation of the software and hardware in a printer makes it difficult to print elaborate complex organs with other delicate structures, such as capillaries, biliary trees, and kidney tubules, mimicking their natural counterparts. It is more difficult to print both hierarchical vascular and neural networks in a 3D construct based on the simple physical/chemical polymer crosslinking principles [145,146,147,148,149] (Figure 9). Especially, most of the chemical crosslinking requires complex modification treatments, and many of the chemical reactions are likely to damage cells. It is reasonable that the more complex an organ is, the more sophisticated are the 3D printers and biomaterials needed to simulate it.

## 5. Conclusions

The use of sacrificial biomaterials has shown great potential in printing complex tissues and organs with hierarchical vascular networks. Especially, the introduction of sacrificial polymers has overcome some bottleneck problems of 3D bioprinting, such as the collapse when printing tubular tissue structures and the inability to print soft biomaterials. Sacrificial polymers can also increase the porosity of printed 3D structures, which are more conducive to cell proliferation, migration, and connection. Most importantly, the sacrificial biomaterials mentioned in this review have already become available from companies such as Sigma-Aldrich, Lubrizol, Fisher Chemical, etc., which is one of the factors allowing them to be used widely and rapidly. This strategy of employing sacrificial biomaterials in 3D bioprinting has provided researchers with new avenues for complex organ manufacturing. Looking to the future, 3D bioprinting will become one of the major enabling technologies for large functional tissue and organ construction with the continuous optimization of the chemical modification and physical properties of sacrificial biomaterials, the development of new bioactive sacrificial biomaterials, the updating of 3D printers and algorithms, and the continuous improvement of printing accuracy. As we have outlined, the use of sacrificial biomaterials may have the potential to change the current paradigm of 3D bioprinting for complex organ manufacturing. Similar to the traditional 3D printing platforms, we believe that the use of sacrificial biomaterials can significantly improve 3D bioprinting research levels.

## Data Availability

Not applicable.
